# Solid pseudopapillary neoplasm of the pancreas in an old man: age does not matter

**Published:** 2012-09-11

**Authors:** Mahdi Bouassida, Mohamed Monji Mighri, Dhouha Bacha, Mohamed Fadhel Chtourou, Hassen Touinsi, Mohamed Msaddak Azzouz, Sadok Sassi

**Affiliations:** 1 Department of Surgery, Mohamed Tahar Maamouri Hospital, 8000 Mrazga, Nabeul, Tunisia; 2 Department of Pathology, Mohamed Tahar Maamouri Hospital, 8000 Mrazga, Nabeul,Tunisia; 3 Department of Gastroenterology, Mohamed Tahar Maamouri Hospital, 8000 Mrazga, Nabeul, Tunisia

**Keywords:** Solid pseudopapillary tumor, pancreas, old, surgery, prognosis

## Abstract

Solid pseudopapillary tumor (SPN) of the pancreas is a rare tumor, but has favorable prognosis. It is typically observed in young women. Only few cases have been reported in young men. We report the observation of a 73-year-old man presented with a palpable mass in the left upper abdomen. CT scan showed 10 cm mass at the tail of the pancreas. This mass had mixed cystic and solid components. The patient underwent a distal pancreatectomy and splenectomy. SPN of the pancreas was diagnosed based on histopathological features. The patient recovered uneventfully and didn't receive adjuvant therapy. A CT scan performed 16 months postoperatively showed no evidence of disease recurrence. Although SPN of the pancreas is typically observed in young women, the diagnosis should not be discounted in old male patients. Male patients and those with old age, atypical histopathology and incomplete resection may have a higher risk of recurrence and death, deserving particular attention.

## Introduction

Solid pseudopapillary tumor (SPN) of the pancreas is a rare tumor that constitutes of 0.2 to 2.7% of the primary non-endocrine tumors of the pancreas. It is usually observed in young women, but it occurs in men in less than 10% of cases, that's why the diagnosis is frequently delayed and tumor size at presentation is frequently large. Due to its rareness and unusual behavior, SPN of the pancreas is often associated with diagnostic and therapeutic challenges. We report the case of a 73-year-old man having a solid pseudopapillary tumor of the tail of the pancreas.

## Patient and observation

**Figure 1:**
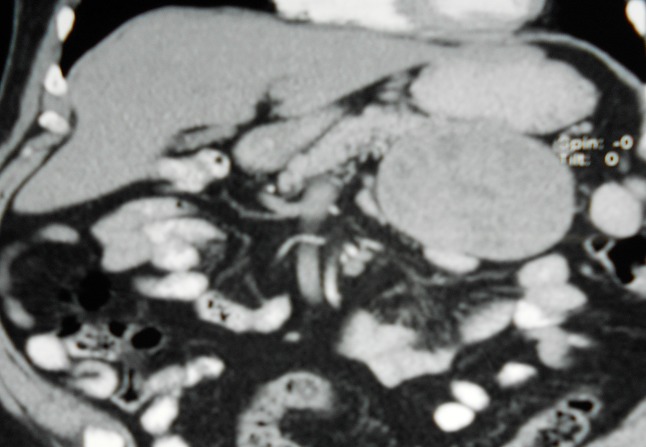
CT scan: well-marginated, encapsulated, heterogeneous mass, at the tail of the pancreas

A 73-year-old male who was in his usual status of good health, presented with intractable vomiting and nausea for two days. He had epigastric pain, increasing abdominal girth of about 4 months duration and 7 kg weight loss over two months. There was no jaundice, change in stool color, diarrhea, or flushing. On examination there was mild diffuse abdominal tenderness in all quadrants, as well as a palpable mass on left upper quadrant. All blood tests were normal, including tumor markers. Ultrasonography was carried out and revealed a 10cm X 8 cm solid-cystic mass in the left upper quadrant of the abdomen. CT scan showed a well-marginated, encapsulated, heterogeneous, hypovascular mass, at the tail of the pancreas, with a thickened, peripherally enhancing capsule. This mass had mixed cystic and solid components (Figure 1).

At laparotomy, there were no metastasis, and no severe adhesion or invasion of the pancreatic mass to the adjacent organs and near-by vessels was grossly noted. A distal pancreatectomy with splenectomy were performed. On macroscopic examination, the tumor was round, fluctuant and measured 10,5x 8x 6,5 cm. It was encapsulated and well-demarcated from the surrounding pancreas. The cut section revealed light brown solid areas with zones of hemorrhage and necrosis at the periphery of the tumor and cystic degeneration centrally.

**Figure 2:**
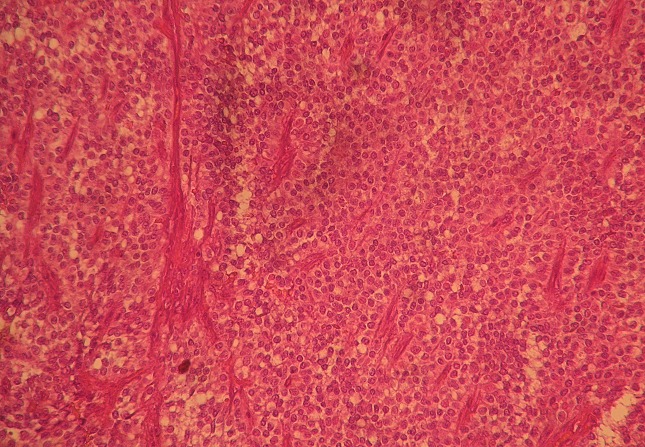
Poorly cohesive monomorphic cells in a solid and pseudopapillary growth pattern (haematoxylin and eosin, x 200)

**Figure 3:**
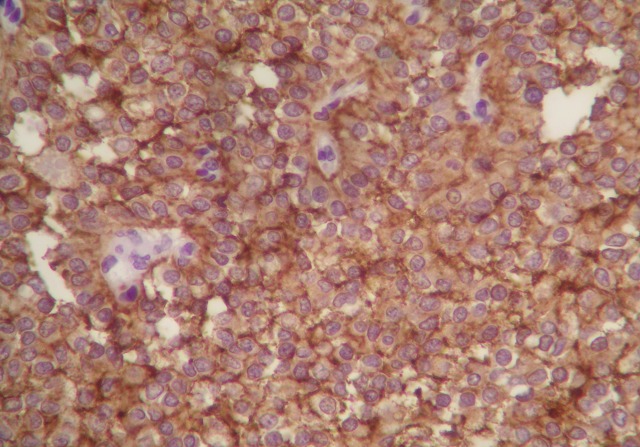
Neoplasic cells stained markedly with CD56 receptors (X 400)

**Figure 4:**
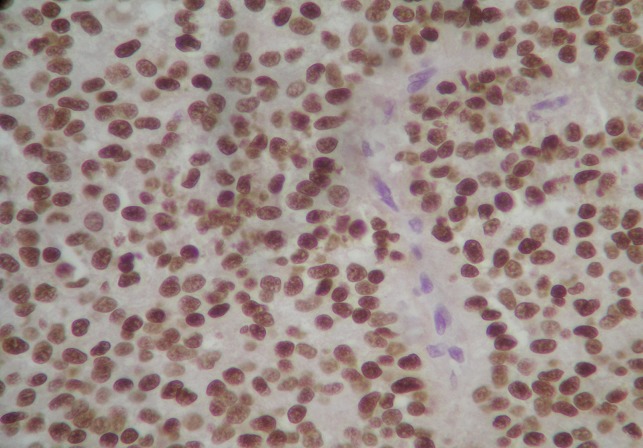
Neoplasic cells stained markedly with progesterone receptors (X 400)

Microscopically, the growth pattern was heterogeneous with a combination of solid, pseudo-papillary and haemorrhagic-necrotic and pseudocystic structures. Solid areas were composed of poorly monomorphic, eosinophilic and clear cells admixed with hyalinized to myxoid stromal bands. Neoplasic cells delicately infiltrated the fibrous capsule, separating the tumor from the normal pancreas (Figure 2). Vascular invasion was rare. Immunohistochemically, tumour cells expressed strongly vimentin, CD10, CD56 and progesterone receptors (Figure 3, Figure 4). There was no expression of antibodies to cytokeratin and neuroendocrin markers (chromogranin and synaptophysin). Four reactive regional lymph nodes were examinated. Spleen specimens were unremarkable. Resection margins were free. These findings were compatible with solid pseudopapillary neoplasm of the pancreas. The patient was subsequently discharged after one week. A CT scan performed 16 months postoperatively showed no evidence of disease recurrence.

## Discussion

SPN has been first described by Franz in 1959 [1] and has been reported under multiple names, including solid and pseudopapillary neoplasm, solid and cystic tumor, papillary cystic tumor, solid papillary epithelial neoplasms, Frantz tumors. It was defined by the World Health Organization (WHO) in 1996 as “solid pseudopapillary tumors” of the pancreas [2]. It is a rare tumor that constitutes of 0.2 to 2.7% of the primary non-endocrine tumors of the pancreas [3].

Typically, SPNs are observed in young women (female: male ratio, 20:1, mean age is 28 years), which supports the ovarian cell theory described above; indeed, over 90% of patients have been reported in women under the age of 35 [4], with only a few cases observed in patients more than 50 years [4]. Hormonal influences appear to play a role in the development and the evolution of SPNs [5], but these neoplasms can arise in men, although men comprise <10% of all patients.

Most patients present with vague symptoms which include abdominal pain, increased abdominal distension, gastric outlet obstruction which are related to tumor compression on the adjacent organs, although almost 30% of the patients with SPN are asymptomatic. Generally, there is no specific clinical syndrome for SPN of the pancreas, and the disease tends to be misdiagnosed, especially in male patients.

Radiologic imaging is an essential part of the diagnosis of pancreatic SPN. In CT scan SPN tumors demonstrate no enhancement of the cystic portions but slight enhancement of the solid portions in the arterial phase and marked enhancement in the portal venous phase [6]. Operative resection is the treatment of choice: pancreatic resection with negative surgical margins is required. The type of pancreatectomy depends on the location of the neoplasm (pancreatoduodenectomy for neoplasms in the pancreatic head, distal pancreatectomy for those in the pancreatic body/tail) and, if technically possible, central pancreatectomy for neoplasms of the proximal body of the pancreas [7]. An aggressive approach to involved adjacent organs should be undertaken with the goal of obtaining negative margins (and RO resection) [4]. An extended lymph node dissection is not required, because lymph node metastases are extremely rare in SPN. Metastasis or tumor recurrence may occur in 10 to 15% of solid-cystic pseudopapillary tumors of the pancreas [8, 9]. The role of adjuvant chemotherapy and radiotherapy is undefined due to limited experience with its use [8].

Despite the locally aggressive features, solid-cystic pseudopapillary tumor of the pancreas has a low-grade malignant potential and tends to have a favorable prognosis, even in the presence of metastatic disease [10]. Overall 5-year survival is as high as 97% in patients undergoing surgical resection [8]. Male patients and those with old age, atypical histopathology (large tumors, diffuse growth, cellular/nuclear atypia, mitotic activity, necrosis, invasion/metastasis) and incomplete resection may have a higher risk of recurrence and death, deserving particular attention [11].

## Conclusion

Solid pseudopapillary neoplasm of the pancreas is a rare low-grade malignant tumor. Although this tumor is typically observed in young women, the diagnosis should not be discounted in old male patients. Surgical treatment alone is the best treatment of solid cystic pseudopapillary tumor of the pancreas; it should be pursued regardless of distant metastasis and size. Total resection guarantees best prognosis.
